# Reduced beta2-glycoprotein I protects macrophages from ox-LDL-induced foam cell formation and cell apoptosis

**DOI:** 10.1186/1476-511X-12-174

**Published:** 2013-11-16

**Authors:** Wei-Lin Wang, Zhen-Xing Meng, Sai-Jun Zhou, Chun-Jun Li, Rui Chen, Lin Lv, Ze-Jun Ma, De-Min Yu, Pei Yu

**Affiliations:** 1 2011 Collaborative Innovation Center of Tianjin for Medical Epigenetics, the Key Laboratory of Hormones and Development (Ministry of Health), Metabolic Diseases Hospital & Tianjin Institute of Endocrinology, Tianjin Medical University, 300070, Tianjin, China

**Keywords:** Reduced beta2-glycoprotein I, Beta2-glycoprotein I, Ox-LDL, Foam cell, Apoptosis

## Abstract

**Background:**

Reduced beta2-glycoprotein I (beta2-GPI) is a free thiol-containing form of beta2-GPI that displays a powerful effect in protecting endothelial cells from oxidative stress-induced cell death. The present study aims to investigate the effect of beta2-GPI or reduced beta2-GPI on ox-LDL-induced foam cell formation and on cell apoptosis and to determine the possible mechanisms.

**Methods:**

The RAW264.7 macrophage cell line was selected as the experimental material. Oil red O staining and cholesterol measurement were used to detect cholesterol accumulation qualitatively and quantitatively, respectively. Flow cytometry was used to detect cell apoptosis. Real-time quantitative PCR was used to detect the mRNA expression of the main proteins that are associated with the transport of cholesterol, such as CD36, SRB1, ABCA1 and ABCG1. Western blot analysis was used to detect the protein expression of certain apoptosis-related proteins, such as caspase-9, caspase-3, p38 MAPK/p-p38 MAPK and JNK/p-JNK.

**Results:**

Beta2-GPI or reduced beta2-GPI decreased ox-LDL-induced cholesterol accumulation (96.45 ± 8.51 μg/mg protein vs. 114.35 ± 10.38 μg/mg protein, *p* < 0.05;74.44 ± 5.27 μg/mg protein vs. 114.35 ± 10.38 μg/mg protein, *p* < 0.01) and cell apoptosis (30.00 ± 5.10% vs. 38.70 ± 7.76%, *p* < 0.05; 20.66 ± 2.50% vs. 38.70 ± 7.76%, *p* < 0.01), and there are significant differences between beta2-GPI and reduced beta2-GPI (*p* < 0.05). Reduced beta2-GPI decreased the ox-LDL-induced expression of CD36 mRNA and ABCA1 mRNA (*p* < 0.05), as well as CD36, cleaved caspase-9, cleaved caspase-3, p-p38 MAPK and p-JNK proteins (*p* < 0.05 or *p* < 0.01). Beta2-GPI did not significantly decrease the expression of ABCA1 mRNA and the p-p38 MAPK protein.

**Conclusions:**

Both beta2-GPI and reduced beta2-GPI inhibit ox-LDL-induced foam cell formation and cell apoptosis, and the latter exhibits a stronger inhibition effect. Both of these glycoproteins reduce the lipid intake of macrophages by downregulating CD36 as well as protein expression. Reduced beta2-GPI inhibits cell apoptosis by reducing the ox-LDL-induced phosphorylation of p38 MAPK and JNK, and the amount of cleaved caspase-3 and caspase-9. Beta2-GPI does not inhibit the ox-LDL-induced phosphorylation of p38 MAPK.

## Background

Foam cells are the characteristic pathological cells in atherosclerotic plaques. The most important reason for foam cell formation is cholesterol accumulation, particularly ox-LDL [1]. A variety of proteins are involved in cholesterol accumulation. ATP-binding cassette transporter A1 (ABCA1) and G1 (ABCG1) are both important outflow pathways for lipids in macrophages [2,3]. The scavenger receptor CD36 is involved in the intake of ox-LDL [4]. In CD36 knockout mice, the capacities of ox-LDL intake and foam cell formation were both significantly reduced [5]. Another scavenger receptor, SRB1, mediates the outflow of cholesterol and inhibits the progress of atherosclerosis [6].

Beta2-glycoprotein I (beta2-GPI) is the main autoantigen for antiphospholipid syndrome, and its molecular weight is approximately 50 kDa, with a circulating concentration of approximately 4 μmol/L in human plasma [7]. Its physiological function remains unclear. Beta2-GPI is composed of five complementary control protein modules, named domain I to domain V, and domain V contains the binding site for negatively charged phospholipids [8]. In the crystal structure of beta2-GPI, a certain disulphide bond is formed between Cys288 and Cys326 of domain V, which is exposed on the surface of this protein [8,9]. This disulphide bond could be opened by thioredoxin-1 (TRX-1), which resulted in the formation of two free thiols. This form of beta2-GPI is called reduced beta2-GPI [10,11].

Beta2-GPI is closely associated with atherosclerosis. George et al. proved the presence of beta2-GPI in atherosclerotic plaques [12]. Lin et al. found beta2-GPI could not only inhibit the translocation of cholesterol from extracellular pools to macrophages but also prevent NO-induced apoptosis in vascular cells [13,14]. Reduced beta2-GPI was recently found to be a protective factor against oxidative stress-induced endothelial cell death [10], which reminded us of its potential effect against oxidative stress. Our preliminary experiments indicated that beta2-GPI or reduced beta2-GPI alone inhibits foam cell formation from U937 human macrophages. The present study aims to investigate the effect of beta2-GPI or reduced beta2-GPI on ox-LDL-induced foam cell formation and cell apoptosis and to determine the possible mechanisms of these effects.

## Results

### Results of Oil red O staining

**Figure 1:**
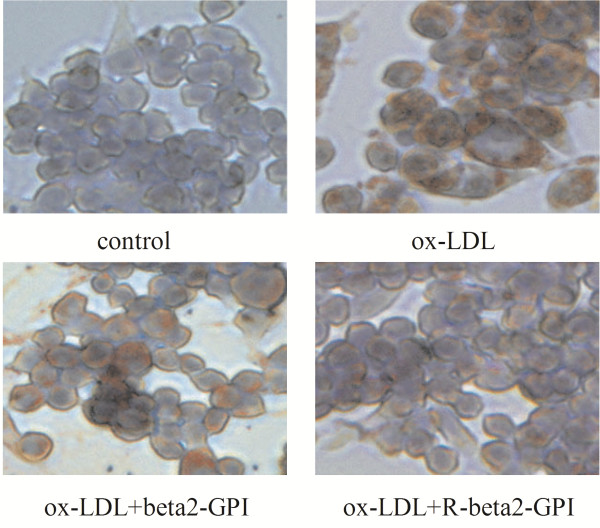
**Typical pictures of macrophages (Oil red O staining, 320×).** RAW264.7 macrophages were seeded onto 96-well microtitre plates at a density of 2 × 10^6^ cells per well, cultured for 12 h and then serum-starved for another 24 h. Cells were incubated for another 24 h in DMEM, which was supplemented with ox-LDL alone or along with beta2-GPI or reduced beta2-GPI. Oil red O staining was performed, and macrophages were observed using an inverted microscope. Ox-LDL increased the formation of red lipid droplets in macrophages and beta2-GPI or reduced beta2-GPI decreased the formation lipid droplets, while the latter showed a stronger action than the former.

In the control group, most of the macrophages had no red lipid droplets, which indicated a low content of the intracellular lipid. In the ox-LDL group, the macrophages increased in size and many red lipid droplets could be clearly observed in the cytoplasm. When beta2-GPI or reduced beta2-GPI was added, red lipid droplets in macrophages decreased, and the latter showed a larger decrease than the former (Figure 1).

### Cholesterol content in macrophages

**Figure 2:**
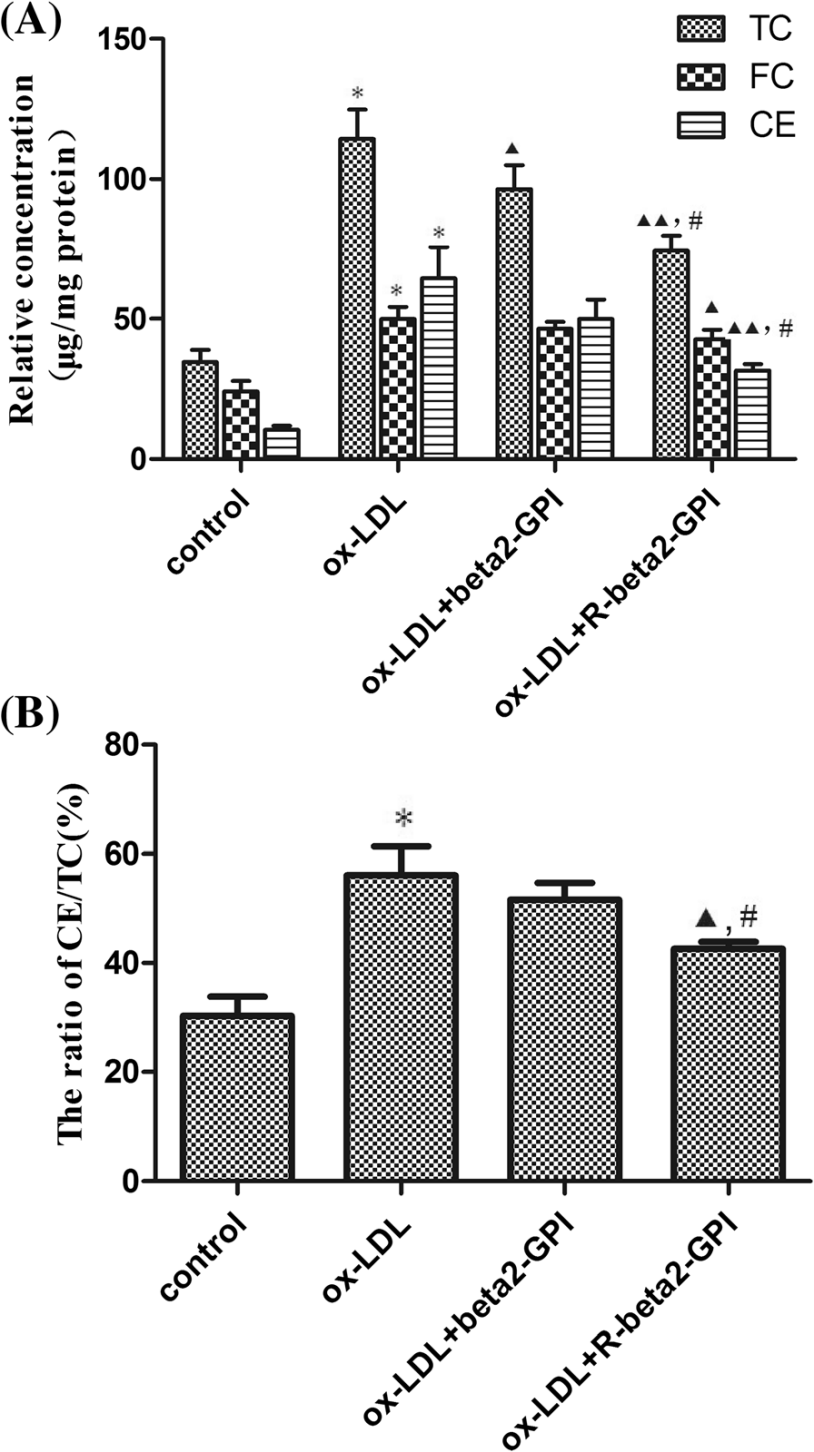
**Intracellular cholesterol content of RAW264.7 macrophage. (A)** The content of intracellular TC, FC and CE. **(B)** The ratio of CE to TC. RAW264.7 macrophages were seeded onto 96-well microtitre plates and then supplemented with ox-LDL alone or along with beta2-GPI or reduced beta2-GPI. HPLC-MS method was used to quantitatively determine the content of free cholesterol. Total protein was detected by the BCA method, and the final cholesterol content was determined using the ratio between the cholesterol concentration and the corresponding protein concentration (μg/mg protein). Ox-LDL increased the content of TC, FC, CE and the value of CE/TC. Beta2-GPI or reduced beta2-GPI decreased TC content in macrophages, whereas latter caused a larger decrease than the former. Note: n = 3, TC, total cholesterol; FC, free cholesterol; CE, cholesterol ester. *: compared with the control group, *p* < 0.01; ▲: compared with the ox-LDL group, *p* < 0.05; ▲▲: compared with the ox-LDL group, *p* < 0.01; #: compared with the beta2-GPI group, *p* < 0.05.

In the ox-LDL group, the contents of TC, FC, CE and the value of CE/TC were all higher than those contents in the control group (*p* < 0.01). The CE/TC value exceeded 50%. Compared with the ox-LDL group, the TC content of cells was decreased in both the ox-LDL + beta2-GPI group and in the ox-LDL + reduced beta2-GPI group (p < 0.05), whereas reduced beta2-GPI caused a larger decrease than beta2-GPI (p < 0.05) (Figure 2A,B).

### Results of flow cytometry

**Figure 3:**
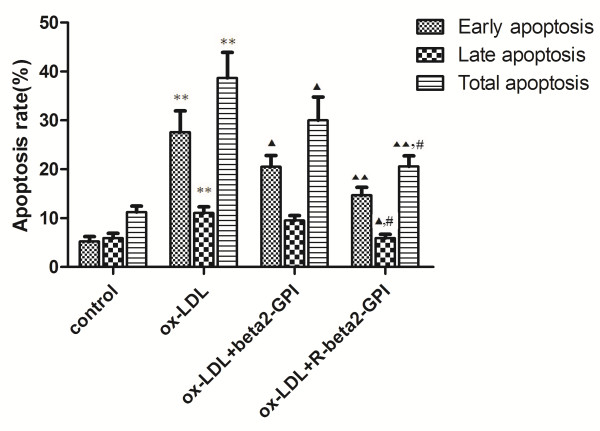
**Apoptosis rate of macrophages.** RAW264.7 macrophages were seeded onto 96-well microtitre plates and then supplemented with ox-LDL alone or along with beta2-GPI or reduced beta2-GPI. Annexin V-FITC/PI double-labelling and flow cytometry were used to detect the apoptosis rate of macrophages. Ox-LDL (75 μg/mL) increased the total apoptosis rate of cells. Beta2-GPI and reduced beta2-GPI significantly decreased the total apoptosis rate of cells, and there was also a significant difference between the two groups. Note: n = 3, *: compared with the control group, *p* < 0.01; ▲: compared with the ox-LDL group, *p* < 0.05; ▲▲: compared with the ox-LDL group, *p* < 0.01; #: compared with the beta2-GPI group, *p* < 0.05.

The total apoptosis rate of macrophages in the blank control was 11.14 ± 3.15%. After incubation with ox-LDL (75 μg/mL) for 24 h, the total apoptosis rate of the cells reached 38.70 ± 7.76%. Beta2-GPI and reduced beta2-GPI significantly decreased the total apoptosis rate of the cells (30.00 ± 5.10% vs. 38.70 ± 7.76%, *p* < 0.05; 20.66 ± 2.50% vs. 38.70 ± 7.76%, *p* < 0.01). In addition, there was also a significant difference between the two groups (20.66 ± 2.50% vs. 30.00 ± 5.10%, *p* < 0.05) (Figure 3).

## PCR results

**Figure 4:**
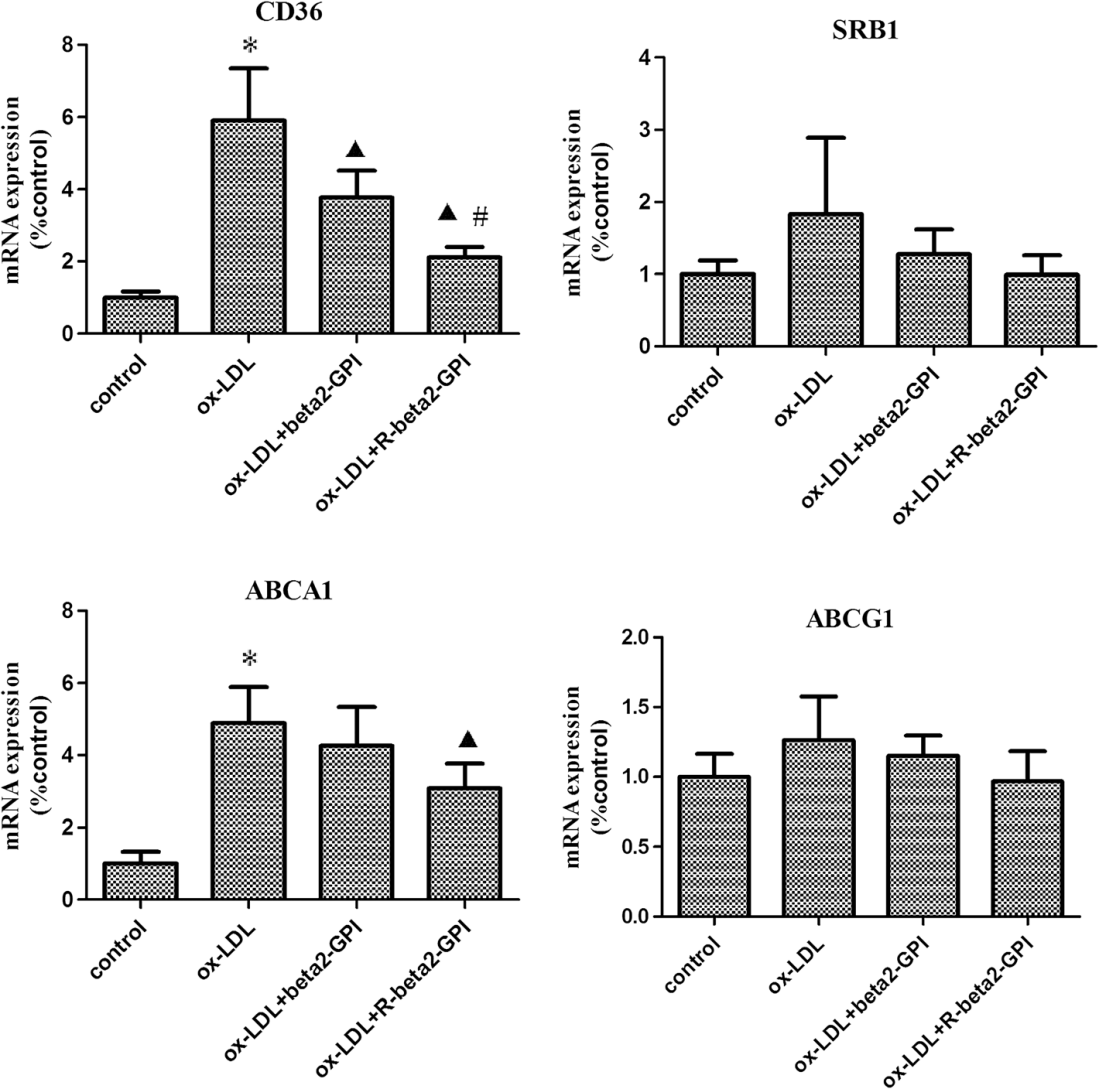
**The expression of CD36, SRB1, ABCA1, and ABCG1 mRNAs in macrophages (compared with control).** RAW264.7 macrophages were seeded onto 96-well microtitre plates and supplemented with ox-LDL alone or along with beta2-GPI or reduced beta2-GPI. Real-time PCR was used to detect the mRNA levels of CD36, SRB1, ABCA1, and ABCG1. β-actin was selected as the internal control. Ox-LDL increased the expression of CD36 and ABCA1 mRNAs. Both beta2-GPI and reduced beta2-GPI inhibited the ox-LDL-induced expression of CD36 mRNA, and there was also a significant difference between the two groups. In addition, reduced beta2-GPI inhibited the ox-LDL-induced expression of ABCA1 mRNA. Note: n = 3, *: compared with the control group, *p* < 0.01; ▲: compared with the ox-LDL group, *p* < 0.05; ▲▲: compared with the ox-LDL group, *p* < 0.01; #: compared with the beta2-GPI group, *p* < 0.05.

Compared with the control group, the expression of CD36 mRNA and ABCA1 mRNA were both increased in cells of the ox-LDL group (*p* < 0.05), whereas the expression of SRB1 mRNA and ABCG1 mRNA showed no significant change. Both beta2-GPI and reduced beta2-GPI inhibited the ox-LDL-induced expression of CD36 mRNA (*p* < 0.05), and there was also a significant difference between the two groups (*p* < 0.05). Reduced beta2-GPI also inhibited the ox-LDL-induced expression of ABCA1 mRNA (*p* < 0.05). There was no significant difference in the expression levels of SRB1 mRNA and ABCG1 mRNA in each group (Figure 4).

### Western blot results

**Figure 5:**
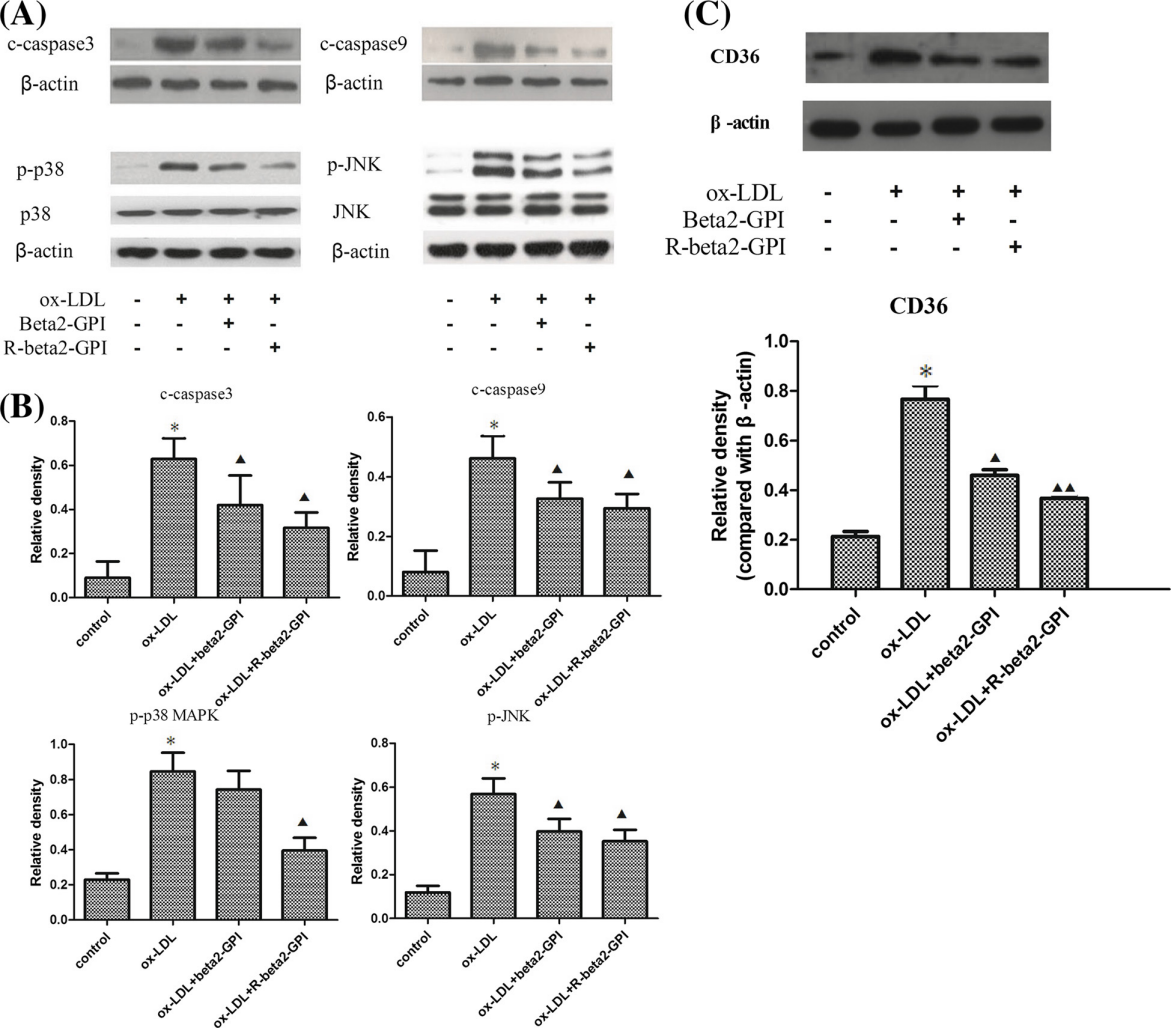
**Relative expression of c-caspase-3, c-caspase-9, p-p38 MAPK, p-JNK and CD36. (A)** Typical western blot bands of c-caspase-3, c-caspase-9, p-p38 MAPK and p-JNK. **(B)** Relative density of c-caspase-3, c-caspase-9, p-p38 MAPK and p-JNK. **(C)** Typical western blot bands and relative density of CD36. RAW264.7 macrophages were seeded onto 96-well microtitre plates and supplemented with ox-LDL alone or along with beta2-GPI or reduced beta2-GPI. Total protein was measured with a Micro BCA^TM^ Protein Assay Reagent Kit. Aliquots of the total protein were resolved by 4–12% Bis-Tris Gel and blotted onto a nitrocellulose transfer membrane. The membrane was incubated with specific primary antibodies and then with HRP-anti-rabbit IgG. Radiography was performed using a UVP analytical instrument. β-actin was selected as the internal control. Ox-LDL increased the expression of c-caspase-3, c-caspase-9, p-p38 MAPK, p-JNK and CD36 in macrophages. Both beta2-GPI and reduced beta2-GPI inhibited the ox-LDL-induced increase of c-casepase-3, c-casepase-9, p-JNK and CD36, and the latter also inhibited the expression of p-p38 MAPK. Note: n = 3, *: compared with the control group, *p* < 0.01; ▲: compared with the ox-LDL group, *p* < 0.05; ▲▲: compared with the ox-LDL group, *p* < 0.01; #: compared with the beta2-GPI group, *p* < 0.05.

Compared with the control group, the expression of CD36, cleaved caspase-3, cleaved caspase-9, p-p38 MAPK and p-JNK in macrophages of the ox-LDL group were all significantly increased (*p* < 0.01). Both beta2-GPI and reduced beta2-GPI inhibited the ox-LDL-induced increase of CD36, cleaved caspase-3, cleaved caspase-9 and p-JNK (*p* < 0.01 or *p* < 0.05), and the latter also inhibited the expression of p-p38 MAPK (*p* < 0.01). There was a significant difference (*p* < 0.05) in the expression level of p-p38 MAPK between the two groups (Figure 5A,B).

## Discussion

The RAW264.7 cell line was established from the ascites of a tumour that was induced in a male mouse by the intraperitoneal injection of Abselon Leukaemia Virus (ATCC Number: TIB-71). RAW264.7 macrophages are easy to culture and have strong adhesion and phagocytosis abilities, which cause its wide use in studies of the pathways of foam cell formation [15,16]. There are two important biological characteristics of foam cells. On one hand, the lipid content in the cytoplasm increases significantly, and lipids are gathered into droplets, which are primarily arranged around the inside surface of the cytomembrane; on the other hand, there is a significant increase in the content of total intracellular cholesterol, and the cholesteryl ester content is over 50% of the total intracellular cholesterol [17]. In the present study, macrophages meet both typical characteristics of foam cells after incubation with 75 mg/L of ox-LDL for 24 h.

To determine the concentration of beta2-GPI and reduced beta2-GPI, we set a concentration gradient (50, 100, 150, 200 μmmol/L) in the preliminary experiment and detected the intracellular total cholesterol. Results revealed that beta2-GPI or reduced beta2-GPI alone inhibited ox-LDL-induced cholesterol influx in a dose-dependent fashion. Results showed a statistical significance using the concentration of 100 μmmol/L beta2-GPI or reduced beta2-GPI. Synthesizes related references [13,14,18,19] and our preliminary experiment, the concentration of 100 μmmol/L was selected for the mechanism study.

Our study demonstrates for the first time that both beta2-GPI and reduced beta2-GPI inhibit ox-LDL-induced foam cell formation and cell apoptosis in vitro, and the latter demonstrates a stronger inhibition effect. Both of these glycoproteins reduce the lipid intake of macrophages by downregulating CD36 mRNA as well as protein expression. Reduced beta2-GPI inhibits cell apoptosis by reducing the ox-LDL-induced phosphorylation of p38 MAPK and JNK, as well as the amount of activated caspase-3 and caspase-9. Beta2-GPI does not inhibit the ox-LDL-induced phosphorylation of p38 MAPK.

Beta2-GPI could combine with atherosclerosis factors, such as ox-LDL, CRP and Lp(alpha), to form corresponding stable complexes [20-23]. Ox-LDL/beta2-GPI complexes were found in patients that suffer autoimmune diseases [24], diabetes [25], etc. Autoantibodies further combined with beta2-GPI in the complexes to form ox-LDL/beta2-GPI/autoantibody complexes (actually antigen/antibody complexes), which could be phagocytised by macrophages, and actually increased ox-LDL intake and foam cell formation [20,24,26]. However, our study revealed that beta2-GPI inhibited ox-LDL intake and foam cell formation in vitro. One possible reason for this result is that there are no autoantibodies in vitro. Thus, beta2-GPI binds with ox-LDL; however, the complexes could not be phagocytised by macrophages. In addition, our study found that beta2-GPI inhibited the ox-LDL-induced expression of CD36 mRNA. Both of these reasons contribute to the decrease in ox-LDL intake and foam cell formation.

Reduced beta2-GPI was first discovered by Ioannou Y et al. in 2010 [10]. Both beta2-GPI (actually oxidised form) and reduced beta2-GPI have recently been found in human serum. Moreover, the proportion of reduced beta2-GPI is significantly lower in the antiphospholipid syndrome group than that in healthy individuals [27], which suggests that reduced beta2-GPI may play a protective role in our bodies. The reduced form of beta2-GPI is generated when the functional disulphide (Cys288-Cys326) is opened, and this change results in some functional changes [10,27]. Reduced beta2-GPI was recently found to protect EAhy926 (human vascular endothelial cells) from oxidative stress-induced endothelial cell damage [15] and to display increased binding to von Willebrand factor (vWF) than non-reduced beta-GPI in vitro [28].

At present, no studies have reported the correlation between reduced beta2-GPI and atherosclerosis. Our study first discovered that reduced beta2-GPI inhibited ox-LDL-induced cholesterol accumulation in macrophages by inhibiting CD36 expression. However, reduced beta2-GPI simultaneously inhibited ABCA1 expression, which is a protein that decreases cholesterol accumulation in macrophages. Because the net result of these changes is the reduction of foam cell formation, it is proposed that there are also other mechanisms in addition to the decrease of CD36 expression that are responsible for the cholesterol efflux that is induced by reduced beta2-GPI.

Both macrophages and smooth muscle cells undergo apoptosis in atherosclerotic plaques [29]. Macrophage apoptosis promotes the development of the necrotic core, which is a key factor in rendering plaques vulnerable to disruption and in acute luminal thrombosis [30]. Both p38 MAPK and JNK signalling pathways play an important role in cell apoptosis [31,32]. Caspase-9 and caspase-3 are key proteins in apoptotic pathways [33]. Our study proved that reduced beta2-GPI inhibited the ox-LDL-induced phosphorylation of p38 MAPK and JNK, as well as the amount of activated caspase-9 and caspase-3, thus inhibiting the apoptosis of macrophage-derived foam cells.

Reduced beta2-GPI provides a new direction for the study of beta2-GPI. Currently, the function of reduced beta2-GPI has been poorly understood. Further studies should be performed to investigate this protein’s physiological and pathological roles and to lay the foundation for further clarify related diseases, such as autoimmune diseases and atherosclerosis, as well as biological processes, such as coagulation and oxidative stress. Reduced beta2-GPI may offer a new target for the diagnosis and treatment of certain diseases in the future.

## Conclusions

Both beta2-GPI and reduced beta2-GPI inhibit ox-LDL-induced foam cell formation and cell apoptosis, and the latter exhibits a stronger inhibition effect. Both of these glycoproteins reduce the lipid intake of macrophages by downregulating CD36 mRNA as well as protein expression. Reduced beta2-GPI inhibits cell apoptosis by reducing the ox-LDL-induced phosphorylation of p38 MAPK and JNK, as well as the amount of cleaved caspase-3 and caspase-9. Beta2-GPI does not inhibit the ox-LDL-induced phosphorylation of p38 MAPK.

## Methods

### Materials and regents

The RAW264.7 macrophage cell line was purchased from the American Type Culture Collection (ATCC number: TIB-71). Dulbecco’s modified Eagle Medium (DMEM), fetal bovine serum and ox-LDL were purchased from Beijing Solarbio Science & Technology Co., Ltd. (Beijing, China). Oil red O and hematoxylin were purchased from Sigma. An annexin V-FITC apoptosis detection kit was purchased from Nanjing KeyGEN Biotech. Co., Ltd. (Nanjing, China). The TRIzol reagent was purchased from Invitrogen (Carlsbad, America). A Reverse Transcription Kit and SYBR ® Premix Ex TaqTM DNA polymerase were purchased from TaKaRa Bio. Inc. (Otsu, Japan). Rabbit anti-mouse monoclonal antibodies to p38 MAPK, p-p38 MAPK, JNK and p-JNK were purchased from Cell Signaling Technology, Inc. (Boston, America). Rabbit anti-mouse monoclonal antibodies to cleaved caspase-3 and cleaved caspase-9 were purchased from Santa Cruz Biotechnology, Inc. (Santa Cruz, America). Horseradish peroxidase-labelled goat anti-rabbit IgG was purchased from Beijing ComWin Biotech Co., Ltd. (Beijing, China).

### Purification of beta2-GPI

Beta2-GPI was purified from normal human plasma by methods that were described previously [14]. Plasma beta2-GPI was precipitated by 3% (v/v) perchloric acid and isolated by heparin-Sepharose affinity chromatography (HiTrap Heparin, GE Healthcare). LC-MS analysis was used to confirm this protein. The purity of beta2-GPI was confirmed by sodium dodecyl sulphate-polyacrylamide gel electrophoresis (SDS-PAGE) on a 10% mini-gel. The SDS-PAGE analysis of the protein sample showed an identical band to that of the standard sample. The BCA method was used to determine the concentration of beta2-GPI.

### Preparation of reduced beta2-GPI

Reduced beta2-GPI was prepared by methods that were described previously [10]. Purified beta2-GPI (1 μM) was reduced by TRX-1 (3.5 μM), which was activated by dithiothreitol (DTT, 70 μM). The thiols of reduced beta2-GPI were protected by reduced glutathione. The reduced beta2-GPI was verified using a Western blot and LC-MS analysis. The Western blot analysis showed that the new protein maintained the same immunological activity with beta2-GPI. The LC-MS analysis verified that domain V of the new protein had free thiols.

### Cell culture

RAW264.7 cells were cultured in DMEM with 25 mmol/L glucose, which was supplemented with 10% (v/v) fetal bovine serum at 37°C in a humidified atmosphere with 5% CO_2_. Cells were routinely subcultured when grown to subconfluency (>90% by visual estimate).

Cells were seeded onto 96-well microtitre plates at a density of 2 × 10^6^ cells per well, cultured for 12 h and then serum-starved for another 24 h. Four groups were designed, and cells were incubated for another 24 h in DMEM, which was supplemented with 25 mmol/L glucose, 10% (v/v) fetal bovine serum and the following intervention factors: (a) blank control; (b) 75 μg/mL ox-LDL; (c) 75 μg/mL ox-LDL + 100 μg/mL beta2-GPI; (d) 75 μg/mL ox-LDL + 100 μg/mL reduced beta2-GPI.

### Oil red O staining

Cell culture was the same as above. Culture medium was removed, and cells were washed three times with PBS and fixed in formalin for 30 minutes. Fixed cells were rinsed with PBS and then with 60% isopropanol for 5 minutes, and then stained with freshly prepared Oil Red O working solution for 30 min at 60°C. The nuclei were lightly stained with haematoxylin for 5 minutes. Stained cells were rinsed with distilled water, mounted in glycerine jelly, and then observed using an inverted microscope.

### High performance liquid chromatography-mass spectrometry (HPLC-MS)

Column: Inertsil ODS column (5 μm, 150 mm × 4.6 mm, purchased from GL Sciences, Tokyo, Japan). Mobile phase: methanol–water (78:22). Flow rate: mL · min^-1^. Temperature: room temperature. Application volume: 10 μL.

A previous study has described the quantitative determination of cholesterol by the HPLC-MS method [34]. Based on this method, we made some changes according to our situation. Cells were washed once with cold PBS and then sonicated in ice bath for 5 min. The cell lysate was then subpackaged (200 μL every part). In total, 300 μL of freshly prepared 15% alcohol-KOH was added to 200 μL of cell lysate. After 2 hours, 500 μL of hexane:isopropanol solution (4:1, v/v) was added. After 15 min vortex-mixing, the cell lysate was centrifuged at 4000 rpm for 5 min, and the upper organic phase was collected. Residual free cholesterol in the lower aqueous phase was extracted twice as described above. Then, the total clarified supernatants were collected and evaporated to dryness by vacuum centrifugation. Stigmasterol was used as an internal standard. A calibration curve was constructed according to the LC-MS analysis of solutions that contained stigmasterol (10 μg/mL) and serial dilutions of cholesterol. Then, the dry sample was dissolved with a stigmasterol solution (10 μg/mL) and analysed using the LC-MS method. The total protein in the aqueous phase was detected by the BCA method, and the final cholesterol content was determined using the ratio between the cholesterol concentration and the corresponding protein concentration (μg/mg protein). To detect the total cholesterol content, cholesterol esterase was used to hydrolyse cholesterol ester to free cholesterol. The content of cholesterol ester was equal to the difference between total cholesterol and free cholesterol.

### Flow cytometry

Cell culture was the same as above. Cells were digested with trypsin and collected using centrifugation (2000 rpm, 5 min). 500 μL binding buffer was added to cells. Then 5 μL annexin V-FITC and 5 μL propidium iodide were added to the binding buffer. Samples were kept in a dark place for 5–15 mins at room temperature and then flow cytometric analysis was performed. The excitation wavelength was 488 nm. The emission wavelength was 530 nm.

### Real-time quantitative PCR

Primers for all tested genes were designed using the Primer 5.0 software and were evaluated using the Oligo 6.0 software. The primer sequences for CD36 were: forward 5'-GAACCACTGCTTTCAAAAACTGG-3, reverse 5'-TGCTGTTCTTTGCCACGTCA-3'; for SRB1: forward 5'-TTTGGAGTGGTAGTAAAAAGGGC-3', reverse 5'-TGACATCAGGGACTCAGAGTAG-3'; for ABCA-1: forward 5'-AGTGATAATCAAAGTCAAAGGCACAC-3'; reverse 5'-AGCAACTTGGCACTAGTAACTCTG-3'; for ABCG1: forward 5'-TTCATCGTCCTGGGCATCTT-3', reverse 5'-CGGATTTTGTATCTGAGGACGAA-3'; for β-actin: forward 5'-TGGAGAAGAGCTATGAGCTGCCTG-3', reverse 5'-GTGCCACCAGACAGCACTGTGTTG-3'.

Total cellular RNA was isolated using the TRIzol reagent according to the manufacturer’s instructions. Final RNA concentrations were determined by OD values at 260 nm, and integrity was verified by ethidium bromide staining of ribosomal 18S and 28S bands on an agarose gel. The A_260_/A_280_ ratios were from 1.8-2.0. Total RNA was reverse transcribed into cDNA using a Reverse Transcription Kit. The PCR reaction system was composed of cDNA, primer, SYBR Green Mix and ddH_2_O. PCR cycling conditions were set as follows: 94°C for 2 min, then 35 cycles at 94°C for 30 sec, 60°C for 30 sec 45, and 72°C for 20 sec. All sample measurements were performed in triplicate. The relative quantification was based on β-actin genes to determine fold-differences in the expression of the target gene. *ΔΔC*_*t*_*-*method was used for the normalisation procedure. The final result was shown by the ratio of 2 ^–**ΔΔ**Ct^ in the experimental group to that in the control group.

### Western blot

The reaction was terminated by adding 1 mL of cold PBS, which contained 100 μM sodium vanadate. The samples were then placed on ice, washed with ice-cold PBS, and lysed in RIPA lysis buffer for 30 mins. Lysates were clarified by centrifugation at 12000 rpm for 15 min at 4°C, and the protein content in the supernatant was measured with a Micro BCA^TM^ Protein Assay Reagent Kit according to the manufacturer’s instructions. Aliquots (30 μg protein per lane) of the total protein were resolved by NuPAGE^TM^ 4–12% Bis-Tris Gel and blotted onto a nitrocellulose transfer membrane. The membrane was blocked with 2% BSA in TBST (20 mM Tris–HCl, pH 7.6, 137 mM NaCl and 0.01% Tween-20) for 1 h at RT, followed by incubation with specific primary antibodies (to CD36, p38 MAPK, p-p38 MAPK, JNK and p-JNK, cleaved caspase-3 and cleaved caspase-9). After washing with TBST, the membrane was reprobed with HRP-anti-rabbit IgG (1:1000) in 2% BSA in TBST for 60 min at RT. Radiography was performed using a UVP analytical instrument. β-actin was selected as the internal standard protein.

### Statistical analysis

The data are expressed as the mean ± SEM. Differences between two groups were analysed using Student’s *t*-test, and differences between multi-groups were analysed using a one-way analysis of variance. When statistically significant differences were found, individual comparisons were made using Tukey’s test. Significance was taken as *p* < 0.05.

## Competing interests

No competing interest exists in the submission of this manuscript.

## Authors’ contributions

W-LW and Z-XM performed the main experiments and drafted the manuscript. S-JZ and C-JL participated in the design of this study, the analytical work of the data and the draft of the manuscript. RC, LL and Z-JM performed some parts of the experiments. DMY and PY conceived of the study, participated in its design and coordination and helped to draft the manuscript. All authors read and approved the final manuscript.
